# Migratory grief: a systematic review

**DOI:** 10.3389/fpsyt.2024.1303847

**Published:** 2024-02-16

**Authors:** Anna Renner, Viktoria Schmidt, Anette Kersting

**Affiliations:** Department of Psychosomatic Medicine and Psychotherapy, Medical Faculty, Leipzig University, Leipzig, Germany

**Keywords:** migratory grief, migration, mental health, grief, loss

## Abstract

**Introduction:**

Migration is often accompanied by interpersonal, material and abstract losses and can be associated with migratory grief. The correlates of migratory grief have not yet been sufficiently addressed in research. This review aims to systematically investigate the relationship between migratory grief and psychopathology, to map the current state of research on this highly relevant topic and to derive relevant implications for the target group.

**Method:**

A systematic literature search of electronic databases (PubMed/Medline, PsycINFO, Web of Science) was conducted up until January 2023. Primary empirical quantitative and qualitative studies with migrants were included that assessed the association between migratory grief and psychopathology, using a specific instrument for migratory grief (quantitative) or named migratory grief as relevant topic (qualitative). Studies that only captured aspects of migratory grief, were not written in English, or were descriptive/non-peer-reviewed publications, were excluded. A quality assessment of all studies included was performed using the Mixed Methods Appraisal Tool. The results were synthesized using a narrative synthesis approach.

**Results:**

All studies (quan. = 4; qual. = 1) were cross-sectional and used convenience samples. The studies had a mean number of 83 participants with a total of N = 487 participants included in the current review. All included studies reported a significant relationship between migratory grief and psychological distress.

**Discussion:**

Despite the quality of the included studies being limited, our results show that there is a link between migratory grief and depression among refugees and migrants. However, there are only few studies in this currently and certainly also in the future relevant field of research, which is why further studies on factors influencing migratory grief as well as associations with other disorders would be desirable.

**Systematic review registration:**

https://www.crd.york.ac.uk/prospero/, identifier CRD42023403448.

## 1 Introduction

Grief is commonly defined as a natural reaction to loss, which becomes less intense over time and enables adaptation to a new reality (1, 2). However, certain circumstances and factors can lead to associated mental disorders such as persistent grief disorder, depression, anxiety or PTSD (3, 4). Migration takes relevance in this context, as migrants^1^ often experience reactions of grief due to the occurrence of traumatic events and losses. While reasons for migration can be diverse, e.g., the search for protection, saving of lives or opening the access to new opportunities, implications may be devastating (6–8). Migrants can suffer a number of losses that are clearly related to the migration experience. In the following, we refer to these losses as “migration-related losses”. These losses may include the loss of relatives and friends (interpersonal losses), of house and income (material losses) but also of status, social role, identity, communication possibilities due to a new language, of a planned future and dreams or simply of the familiar surroundings (abstract losses) (8, 9). Although losses are such a common and presumably consequential sequel of migration, they receive little attention in research. This applies particularly to abstract losses that are often not socially recognized (9–11). Eisenbruch (12) named the distress occurring after loss of homeland, identity and social connectedness due to relocation “cultural bereavement”. Achotegui (7) developed the concept of the “Ulysses syndrome” to depathologize the feelings of alienation and stress after migration. Nevertheless, we know that the migration experience is associated with increased prevalence of mental distress. A Meta-analysis by Lindert et al. (13) show prevalence rates of 20% for depression among labor migrants and 44% among refugees as well as 21% for anxiety among labor migrants and 40% among refugees. According to a systematic review by Kokou-Kpolou et al. (14), the pooled prevalence of prolonged grief disorder was 33.2% in adult refugees. A number of risk factors for the mental health of migrants have already been investigated, such as gender, income, language skills, social support and social isolation, discrimination (e.g. regarding housing or employment) and legal status (15–18). It is also discussed that vulnerabilities often do not result from independent risk factors, but from intersectional inequalities based on gender, age, race, ethnicity, social class and legal status (19, 20). Although a number of risk factors for migrants’ mental health are associated with various dimensions of loss (17), the processing of loss, i.e. grief, is rarely the focus of research. In addition, since both migration and grief seem to be risk factors for mental disorders, the question arises as to why there is so little research on the specific combination of these two factors and their implications for the mental health of migrants. Therefore, we have set the aim for this review to systematically investigate the relationship between MG and psychopathology (PP) in migrants to clarify its relationship, identify possible research gaps and derive clinical implications.

## 2 Methods

The systematic review was conducted and reported following the PRISMA Statement to the best of ability (21). It was registered with PROSPERO (CRD42023403448).

### 2.1 Search strategy

A systematic literature search of electronic databases (PubMed/Medline, PsycINFO, Web of Science) was conducted without time or language restrictions to identify relevant studies (final search date: 30/01/23). The search term focused on the link between MG and PP: [(psychopathol* OR depression OR psychiatr* OR depressiv* OR “posttraumatic stress” OR “psychological distress” OR “mental health”) AND (displacement OR migratory OR migration OR refugee OR “asylum seeker”) AND (grief OR loss OR mourn* OR “bereavement”)] NOT (cell migration OR erythema). The „snowball-method” was used to identify additional studies, since a hybrid search strategy increases the probability of identifying all relevant studies (22). Citavi 6 was used to catalogue all identified records and remove duplicates. Two authors (AR and VS) screened abstracts independently. After calculation of agreement and discussion with Supervisor AK regarding inconsistencies in inclusion, AR conducted the full text screening for inclusion or exclusion. VS assisted with full text analysis for individual studies where ambiguity was prevalent. Disagreements were resolved by discussion with AK. Using Cohen`s Kappa statistic, κ (23), inter-rater reliability was assessed and equalled 0.929.

### 2.2 Eligibility criteria

Primary empirical quantitative and qualitative studies were included in this review. No time limit was set regarding the publication year of the studies up until the latest search in January 2023. Inclusion criteria were: (1) Participants: Persons of all genders and ages that were exposed to any kind of migration experience (e.g. labor migration, internal displacement, refugees). (2) Exposure: In the included quantitative studies, MG had to be assessed with a specific instrument. In qualitative studies, MG had to be named as such or as a synonym (i.e. cultural bereavement, “Ulysses-syndrome”), (3) Outcomes: The association between MG and PP had to be reported. Excluded were studies that (1) only captured aspects of MG (e.g. interpersonal losses only), (2) were not written in English, (3) were case studies, reviews, descriptive studies, expert opinions, clinical guidelines, conference papers or non-peer-reviewed publications.

### 2.3 Data collection and analysis

When studies met inclusion criteria, the following data was double extracted independently by AR and VS from quantitative studies: (1) Source: author, publication, year, country, journal; (2) Sample: type of sample, sample size, recruitment; (3) Measurements of MG and PP; (4) methods; (5) Outcomes: Correlation coefficients between MG and Psychopathology were extracted from each included study. Bullet points 1-2 were also collected in qualitative studies, in addition to reports on the occurrence of MG in the population and mention of the association with psychological distress.

Results were synthesized narratively and additionally summarized in a table for better overview. Due to the low number of quantitative studies and the heterogeneity in measurement instruments, no meta-analysis was performed.

### 2.4 Quality assessment

The Mixed Methods Appraisal Tool (MMAT) (24) was applied to appraise the quality of quantitative and qualitative studies independently by two researchers. The MMAT was chosen for its acknowledged reliability in evaluating mixed methods research (25). It is essential to highlight that the MMAT does not primarily assess the overall ‘quality’ of a research paper. Rather, it is employed to ascertain whether a study’s methodology meets predefined criteria indicative of methodological quality. Ratings depend on fulfilling criteria, with variations based on the study type. A score of 1 is assigned for each fulfilled criterion, allowing for a maximum total of 4 for each study. If criteria are unmet or information is absent in the study, a score of 0 is assigned. AR and VS independently performed a quality assessment of included studies; disagreements were resolved by discussion with AK. A detailed description of the MMAT rating criteria and the categorization of the included studies can be found in **Appendix 1.1**.

## 3 Results

**Figure 1:**
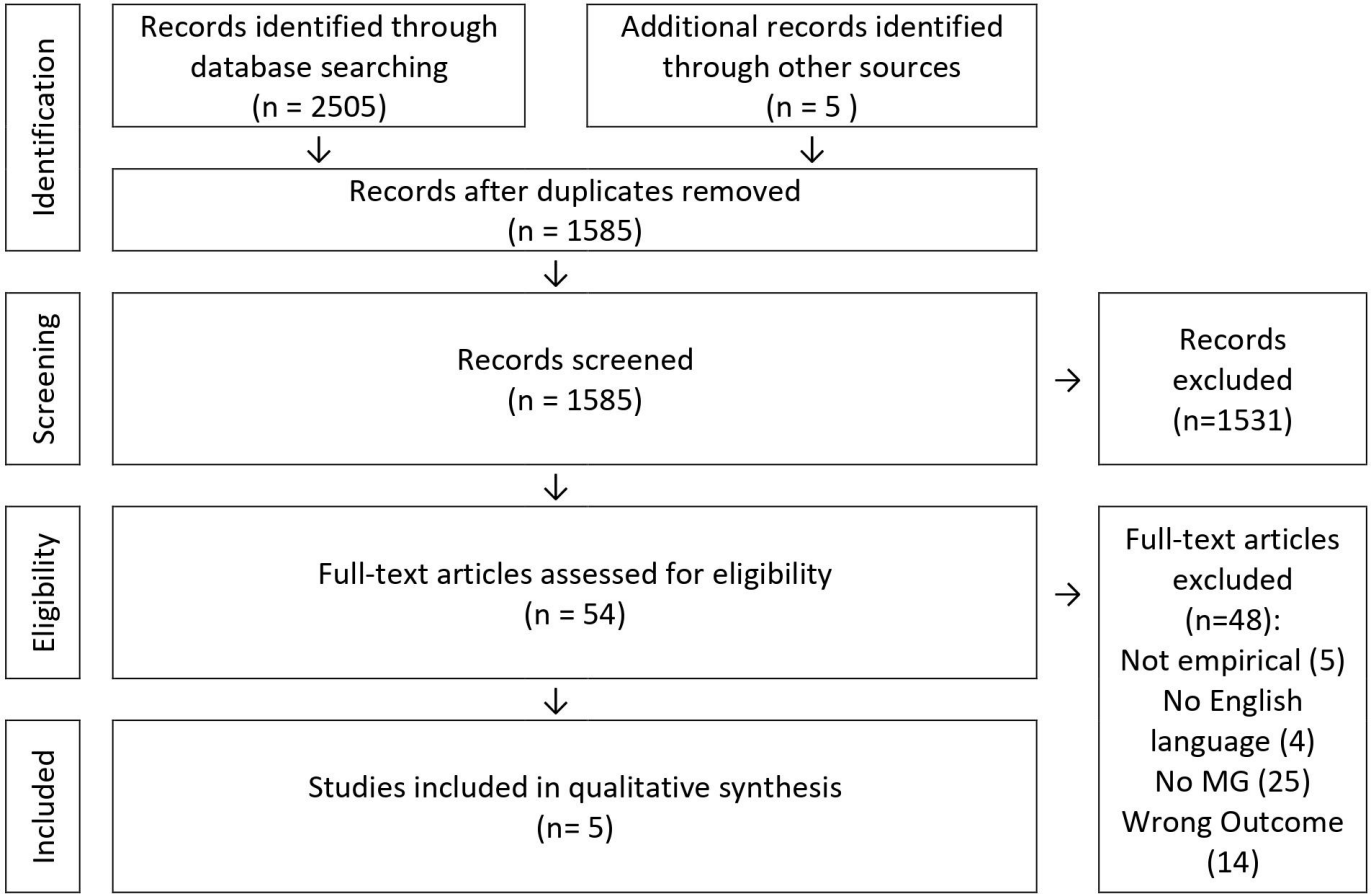
Study selection following PRISMA guidelines (26).

The systematic search yielded a total of 2505 results. After removal of duplicates, 1585 records remained for abstract screening. Of those, 54 records were found suitable for full-text screening and five were included in the current review (4 quantitative and 1 qualitative study). The selection process is pictured in **Figure 1**.

### 3.1 Study characteristics

The included studies examined the association of MG and PP in samples ranging from N=16 (plus 6 family interviews, not numerically included since exact number of participants remains unclear) (27) to N=150 (9, 28) with a total of N=487 participants included in the current review. **Table 1** provides an overview of all included studies. Four studies were conducted in the United States (9, 27–29), one in Australia (30). The target groups included elderly Chinese, Buthanese, or Kurdish refugees and South African immigrants. All studies were cross-sectional and used convenience samples.

**Table 1:** Included studies of MG and PP in alphabetical order.

Author	Design	N	Country of Arrival	Sample			Measurement MG	Measurement PP	Methods	MG/PP Bivariate	MG/PP Multivariate
				Country of Origin / Ethnicity	Reasons for Migration	Average Length of stay in Country of Arrival					
**Casado et al., 2010 (****9****)**	Quanti- tative, cross-sectional	150	USA	Chinese	Political: 5.1% Education: 5.1% Family: 28.6% Better life/opportunities: 52.0% Economic/work: 5.1% Other: 4.1%	M = 10.9 SD = 7.4	MGLQ	Chinese Depressive Symptom Scale-16 (CDS-16)	Exploratory factor analysis, correlation analysis	Significant relation between MGLQ overall score and depression (r = .54, p <.001)	Not investigated
**Casado & Leung, 2002 (****28****)**		150	USA	Chinese	Political: 3.3% Education: 4.0% Family: 28.0% Better life/opportunities: 40.0% Economic/work: 3.3% Other: 3.3% Missing: 18.0%	M = 10.86 SD = 7.39	MGLQ	Chinese Depressive Symptom Scale-16 (CDS-16)	Stepwise regression	Significant relation between MGLQ overall score and depression (r = .65, p <.001)	MG was a significant predictor for depression and contributed to 41.5% of the variance (B = .350; p < .001)
**Cummings et al., 2011 (****29****)**		70	USA	Kurdish	Refugees: 71.4% Asylum seekers: 22.9% Other immigrants: 5.7%	M = 12.8 SD = 3.5	MGLQ	Geriatric Depression Scale (GDS)	Multivariate linear regression	Significant relation between MGLQ overall score and depression (r = .24, p =.05); MGLQ subscales: significant association of subscale "Disorganization" and depression (r = .34, p =.01)	Subscale "Disorganization" was a significant predictor for depression (B = 0.23, p < .05)
**Khawaja & Mason, 2008 (****30****)**		101	Australia	South African (mainly white people)	Violence in South Africa * Job opportunities in South Africa/Austraulia*	Inclusion criterion: <5 years	Grief and Migration Scale	Hopkins Symptom Checklist (Anxiety / Depression)	Multiple regression analysis	Not reported	MG was the strongest predictor for distress, accounting for 39.8% of the variance (B = 2.61; p < .01)

*No percentages detectable in the publication.

### 3.2 Study quality

All included studies were rated regarding methodological quality applying the MMAT. The quantitative studies showed to be of medium quality ranking between a total score of 3 (9, 30) and 4 (28, 29) out of 6. The qualitative study by Im and Neff (27) scored highest with 6 out of 6. Methodological shortcomings were found in the studies, particularly with regard to detailed definitions of the target groups and the complete outcome data. Khawaja and Mason lacked a validated instrument for recording MG. Details of the quality appraisal can be found in **Appendix 1.1**.

### 3.3 Operationalization of MG

All quantitative studies used standardized scales to assess MG and PP. The Migratory Grief and Loss Questionnaire (MGLQ; 9) was applied by three of the four quantitative studies (9, 28, 29). The scale was designed to measure the experience of grief and loss associated with immigration. It captures the dimensions searching and yearning, idealization, and identity discontinuity and consists of 20 items and a 4-point Likert scale (0 = never, 1 = occasionally, 2 = often, 3 = always). Participants are asked how each item applied within the last 30 days. Higher scores indicate higher levels of MG (range 0-60). The fourth quantitative study by Khawaja and Mason (30) used the Grief and Migration Scale to assess immigration-related grief. The instrument is an adaption of the Core Bereavement Scale by Burnett et al. (31) created to capture grief after interpersonal losses. The authors selected fitting items from the scale and adapted the wording to capture the experience of grief for a country rather than a person. The new scale consists of six items and a Likert-type scale ranging from 1 = never to 5 = always. The qualitative study (30) applied an interview guide that explicitly avoided theoretical terms like cultural bereavement with the aim to gather unbiased information in the participants’ own words. Instead, the experience of the participants was asked very broadly focusing on the topics of differences between the countries, adjustment to the host country and cultural challenges and solutions for the migrated community.

The operationalization of Psychopathology can be found in **Appendix 1.2**.

### 3.4 Association of MG and PP

#### 3.4.1 Bivariate analysis

Three out of four quantitative studies reported a bivariate relation between MG and PP (9, 28, 29). Three studies found a negative relationship between MG and depression in elderly Chinese or South African immigrants (r = .54, p <.001; r = .65, p <.001; r = .24, p = .05; 9, 28, 29). Moreover, depression was significantly associated with the „Disorganization” subscale of MG (r = .34, p =.01), but not the other two subscales (29). Khawaja and Mason (30) did not report a bivariate association of MG and PP.

#### 3.4.2 Multivariable analysis

A multivariate analysis of the association between MG and PP was carried out in the studies by Casado and Leung (28), Cummings et al. (29) as well as Khawaja and Mason (30). The MG overall score was identified as a significant predictor for depression and contributed to 41.5% of the overall variance (B = .350; p <.001) ^2^ in Casado and Leungs’ publication (2002). Despite a significant bivariate association of the MGLQ total score with depression, Cummings et al. (29) only included the bivariate significant subscale “Disorganization” in the multivariate analysis. This same subscale showed to be a significant predictor for depression (B = 0.23, p <.05). Khawaja and Mason (30) reported MG as the strongest predictor for emotional distress, accounting for 39.8% of the variance (B = 2.61; p <.01).

#### 3.4.3 Qualitative analysis

The qualitative analysis of Im and Neff (27) showed the following association between MG and PP: After migration, the target group of Hindu Buthanese refugees showed culture shocks due to the shift of living environment from a collective to individualistic society. According to the authors, the resulting experience of loss and grief led to cultural trauma and bereavement, which again led to mental distress. The authors indicated that a loss spiral would be set in motion by these processes, since mental distress would weaken coping capacities.

## 4 Discussion

### 4.1 Summary of key findings

Migration is a global phenomenon as old as human history and its relevance continues to increase due to ongoing conflicts as well as climate change and its consequences (32–34). Losses experienced in the context of migration and the associated mourning must therefore be considered a highly relevant topic. The aim of this systematic review was to provide an overview of available research on the association of MG and PP. Our results show that there is a blatant lack of studies investigating this association. After a comprehensive systematic search, we were able to include five studies, four of which applied a quantitative and one a qualitative design. In summary, all included studies reported a relationship between MG and psychological distress. Three studies reported an association between MG and depression and two studies reported an association between MG and emotional/mental distress. It has to be noted that the target group of four of the five studies consisted of migrants or refugees from the Asian continent to the US. While the samples are unquestionably diverse, it can be generalized that MG occurs among migrants and refugees from the Asian continent in the USA and seems to be related to psychological distress. This is an important finding, since Asian immigration has been an essential part of immigration to the US since the mid-19th century and people from Asia currently form one of the largest groups of immigrants to the US (35, 36).

### 4.2 Research gaps and potential for future research

Although various losses are discussed in the migration and mental health literature (37–41), the bridge to mourning and its impact on mental health is seldom built. Grief is commonly associated with interpersonal losses exclusively, which is probably why MG has so far received little attention in research as well as in the treatment of persons with migration experiences. Consequently, the relationship between MG and PP is even less studied. The studies included in this review exclusively examined the association of MG with depression or a more general measure of emotional distress. Studies indicated that purely interpersonal losses in refugees and migrants may also be related to PTSD and prolonged grief disorder (14). Since MG may require similar processing mechanisms as interpersonal losses (9), examining the relationship of MG to these same constructs would be desirable in future research, as associations can be presumed. Cummings et al. (29) discussed age and a lack of access to cultural and religious institutions for persons from smaller immigrant communities such as the Kurds as important influencing factors regarding their coping in the country of arrival.

### 4.3 Implications

In addition to the identification of necessary research in the field of MG, practical implications can also be derived from the results of the present review. Unfavorable environmental factors can complicate grieving processes and thus make the development of mental disorders more likely (42). At the policy level, it would therefore be desirable to create a welcoming structure in countries of arrival that enables migrants and refugees to access their resources and work through grief processes without being hindered in processing and adaptation by long asylum processes or difficult housing conditions. This can not only prevent suffering, but also avoid additional costs to the health care system in the longer term (43). At the clinical level, MG should be considered in the treatment of refugees and migrants. In the treatment of depression or other PP in refugees and migrants, MG should be considered as a possibly important influencing factor and processing should be supported. In order to be able to adequately support mourning processes, treatment providers should take sociocultural factors into account while avoiding stereotyping or the premature pathologizing of migration-related mourning processes (44–47). For example, Asian Americans are often referred to without specifying to which of the many heterogeneous groups they belong and which specific stressors and needs should be taken into account to provide effective mental health services (48).

### 4.4 Limitations

The results of this review were subject to some limitations that should be considered. On eligibility, the quality of the included studies is limited. All studies used a snowball- or convenience-sampling method, hence the participants are not representative of the target population. Not all studies reported inclusion- and exclusion criteria clearly. In one study, a MG measure was adapted without testing validity and reliability. Studies were not comprehensively controlled for confounders. Especially structural factors and their interaction, such as legal status and various facets of discrimination might complicate mourning processes and should therefore be analyzed as influencing factors on MG and its association with PP. Further, no cut-offs of MG were reported, therefore it was not possible to assess at which point MG values are considered high. Although it can be assumed that MG changes in the course of the stay in the country of arrival (9), only cross-sectional and no longitudinal studies were conducted. Regarding our methodological approach, it should be noted that we only included studies in English, which may have led to the neglect of relevant studies in other languages. Only peer-reviewed studies were included, which may have led to publication bias but also increased the adherence to scientific quality criteria. It should also be borne in mind that the target group of this review includes both migrants and refugees worldwide, which is a very heterogeneous sample and therefore inaccurate generalizations should be avoided. Moreover, only studies with the focus of MG were included, excluding other potential overlapping constructs, e.g., homesickness. However, despite an overlap in content in both constructs (see 49), no discussion has taken place about delineating the constructs or benefiting from the results from the other areas. Furthermore, we excluded studies that covered only partial aspects of MG in order to make a careful operationalization of the concept more likely. However, this may have resulted in the loss of information about the association of aspects of MG (e.g. loss of status) with psychopathology. Nonetheless, this review provides an important contribution, as, to our knowledge, it is the first systematic review to address the highly relevant topic of migratory grief and to demonstrate its link with psychological distress. Furthermore, it highlights the blatant research gap in this area as well as potential approaches for further studies.

## 5 Conclusion

This review provides evidence that there is a link between MG and PP among refugees and migrants. However, there are only a few studies in this field of research, which is currently relevant and will presumably be of great relevance in the future, which is why further studies on factors influencing MG as well as correlations with other disorders would be desirable.

## Data availability statement

The original contributions presented in the study are cited in the article/**Supplementary Material**. Further inquiries can be directed to the corresponding author.

## Author contributions

AR: Conceptualization, Data curation, Investigation, Methodology, Project administration, Visualization, Writing – original draft. VS: Conceptualization, Data curation, Investigation, Writing – review & editing. AK: Resources, Supervision, Validation, Writing – review & editing.

## Footnotes

1 “Migrants” is a broad term that we use in this article to refer to persons with their own experience of migration, i.e. moving away from the place of usual residence for work or education as well as asylum within a country or across international borders (5). As the specific subgroups can be exposed to very different experiences, they are referred to as such in the relevant contexts.

2 B = Unstandardized regression coefficient.
