# Supplementary material for: Migratory grief: a systematic review
Source: Front Psychiatry. 2024 Feb 16;15:1303847. doi: 10.3389/fpsyt.2024.1303847 (PMC10904569; doi:10.3389/fpsyt.2024.1303847)
Supplement: Supplementary file 1 [file DataSheet_1.pdf]

1. Appendix

1.1. Quality Assessment

**Screening Questions**	1. Are there clear research questions?
	2. Do the collected data allow to address the research questions?
**Quantitative non-randomized Studies**	1. Are the participants representative of the target population?<br>a. Does the study report inclusion and exclusion criteria?<br>b. Does the study report non-responders?<br>c. Does the study report whether recruitment was representative?
	2. Are measurements appropriate regarding both the outcome and intervention (or exposure)?<br>a. Are the measurements clearly defined and accurately measured?<br>b. Are measurements justified and appropriate for answering research question?<br>c. Do measurements reflect what they are supposed to measure?<br>d. Were the measures used validated and reliability tested?<br>e. Is the outcome of interest?
	3. Are there complete outcome data?
	4. Are the confounders accounted for in the design and analysis?
**Qualitative Study**	1. Is the qualitative approach appropriate to answer the research question?<br>2. Are the qualitative data collection methods adequate to address the research question?<br>3. Are the findings adequately derived from the data?<br>4. Is the interpretation of results sufficiently substantiated by data<br>5. Is there coherence between qualitative data sources, collection, analysis and interpretation?

Appendix Table 1. Detailled criteria of the Mixed Methods Appraisal Tool Version 2018 [1] applied in this review.

Scoring of the included studies using MMAT Version 18

A score of 1 is assigned for each fulfilled criterion, allowing for a maximum total of 6 for each study. If criteria are unmet or information is absent in the study, a score of 0 is assigned.

Study	Screening questions	Are the participants representative of the target population?	Are measurements appropriate regarding both the outcome and intervention (or exposure)?	Are there complete outcome data?	Are the confounders accounted for in the design and analysis?	Total Score
**Casado et al. (2010) [2]**	1+1	0	1	0	0	3
**Casado et al. (2002) [3]**	1+1	0	1	0	1	4
**Cummings et al. (2011) [4]**	1+1	0	1	0	1	4
**Khawaja & Mason (2008) [5]**	1+1	0	0	0	1	3

Appendix Table 2. Quality Assessment of Included Quantitative Studies using MMAT Version 18.

Study	Screening questions	Is the qualitative approach appropriate to answer the research question?	Are the qualitative data collection methods adequate to address the research question?	Are the findings adequately derived from the data?	Is the interpretation of results sufficiently substantiated by data?	Is there coherence between qualitative data sources, collection, analysis and interpretation?	Total Score
**Im & Neff (2021) [6]**	1+1	1	1	1	1	1	6

Appendix Table 31. Quality Assessment of Included Qualitative Study using MMAT Version 18.

1.2. Operationalization of Psychopathology

Three out of four quantitative studies exclusively used depression measures to capture PP [2;3;4] and one study [5] implemented a measure for emotional distress. The studies by Casado et al. [2] and Casado and Leung [3] assessed depression applying the Chinese Depressive Symptom Scale 16 (CDS-16). The CDS-16 is a scale derived from the Center for Epidemiological Studies-Depression Scale, which contains 6 additional items for better cultural validity [7]. The 16-item-instrument consists of a 4-point Likert scale (1 = always, 2 = from time to time, 3 = occasionally, 4 = never) with a total range of 0-48 with a higher score indicating a higher level of depression. The study by Cummings et al. [4] implemented the Geriatric Depressen Scale (GDS) developed by Yesavage et al. [8]. The instrument consists of 30 dichotomous items for better comprehensibility even with slight cognitive impairments, with a higher score indication higher depression levels. The Hopkins Symptom Checklist [9] was used by Khawaja and Mason [5] to assess symptoms of depression, anxiety, somatisation, obsessive-cumpulsive and interpersonal sensitivity. The instrument consists of 58 items and a 4-point Likert scale (0 = not at all to 4 = extremely) with a higher score indicating higher levels of emotional distress. In the qualitative study by Im and Neff [6], PP was also not explicitly addressed in the interview guide in order not to influence the participants' answers in advance.

2. References

1. Hong QN, Pluye P, Fàbregues S, Bartlett G, Boardman F, Cargo M, et al. Mixed Methods Appraisal Tool (MMAT): version 2018. Canadian Intellectual Property Office, Industry Canada, Montreal (2018).
2. Casado BL, Hong M, Harrington D. Measuring migratory grief and loss associated with the experience of immigration. Res Soc Work Pract (2010) 20(6):611–20. doi: 10.1177/1049731509360840
3. Casado BL, Leung P. Migratory grief and depression among elderly Chinese american immigrants. J Gerontological Soc Work (2002) 36(1-2):5–26. doi: 10.1300/ J083v36n01_02
4. Cummings S, Sull L, Davis C, Worley N. Correlates of depression among older Kurdish refugees. Soc work (2011) 56(2):159–68. doi: 10.1093/sw/56.2.159
5. Khawaja NG, Mason L. Predictors of psychological distress in South African immigrants to Australia. South Afr J Psychol (2008) 38(1):225–46. doi: 10.1177/ 008124630803800112
6. Im H, Neff J. Spiral loss of culture: cultural trauma and bereavement of Bhutanese refugee elders. J Immigrant Refugee Stud (2021) 19(2):99–113. doi: 10.1080/15562948.2020.1736362
7. Lin N. Measuring depressive symptomatology in China. J nervous Ment Dis (1989) 177(3):121–31. doi: 10.1097/00005053-198903000-00001
8. Brink TL, Yesavage JA, Lum B, Heersma P, Adey. Depressive symptoms and depressive diagnoses in a community population. Arch Gen Psychiatry (1982) 45:1078–84.
9. Derogatis LR, Lipman RS, Rickels K, Uhlenhuth EH, Covi L. The Hopkins Symptom Checklist (HSCL): a self-report symptom inventory. Behav Sci (1974) 19 (1):1–15. doi: 10.1002/bs.3830190102
